# Supplementary material for: Voluntary locomotion induces an early and remote hemodynamic decrease in the large cerebral veins
Source: Neurophotonics. 2025 Mar 24;12(Suppl 1):S14609. doi: 10.1117/1.NPh.12.S1.S14609 (PMC11931294; doi:10.1117/1.NPh.12.S1.S14609)
Supplement: Supplementary file 1 [file NPh_012_S14609_SD001.pdf]

Appendix A: Statistical Reports

Statistics Reports (SR1-SR7): The following tables report the mean, standard deviation, type of statistical test, test statistics, degrees of freedom and p-value for each statistical test reported in the main manuscript.

SR1: Figure 2: Spontaneous locomotion induces a fast, remote decrease in the large cerebral draining vein that precedes the regional HbT influx. The three level comparisons (vessel type: artery, whisker vein, draining vein) for 2c-e were subject to a linear mixed model analysis (lmer package RStudio), with vessel type entered as the fixed factor and animal ID as the random factor (to account for variations between groups being driven by a single outlier animal). To further investigate where the specific significant differences were pairwise comparisons (with correction for multiple comparisons) were conducted using the Tukey method (emmeans function RStudio). The two group comparison for 2f was first subject to a Shapiro-Wilk test (shapiro.test function RStudio) to assess normality, and due to a significant result indicating data was not normally distributed, compared using the non-parametric Wilcoxon-Signed ranks test for paired data (wilcox.test function in RStudio, paired = true).

**Figure**	**Mean**	**SD**	**Test**	**Test Statistic**	**Mean Square**	**Degrees of freedom**	**P Value**
2c (Hbt max peak)	Art: 7.33 WV: 3.61 DV: 2.06	Art: 4.77 WV: 2.89 DV: 2.40	Linear mixed model (fixed factor: vessel type)	F = 1668.1	16991	2	p<2.2e-16<br>**Pairwise** art-dv: p<.0001 art-wv: p<.0001 dv-wv: p<.0001
2d (Hbt time to max peak)	Art: 3.13 WV: 3.19 DV: 3.22	Art: 0.96 WV: 1.03 DV: 1.27	Linear mixed model (fixed factor: vessel type)	F=4.51	5.06	2	p=0.011<br>**Pairwise** art-dv: p=0.011 art-wv: p=0.082 dv-wv: p=0.726
2e (Hbt minimum peak)	Art: -0.97 WV: -1.06 DV: -2.77	Art: 2.01 WV: 1.73 DV: 2.56	Linear mixed model (fixed factor: vessel type)	F=582.64	2414.1	2	p<2.2e-16<br>**Pairwise** art-dv: p<.0001 art-wv: p=0.291 dv-wv: p<.0001
2f (compare peak timing (max for art, min for dv)	Art: 3.13	Art: 0.96	Paired Wilcoxon-Signed Rank	W=269		1	p<2.2e-16

	DV: 1.04	DV: 0.94					

SR2: Figure 3: The amount of locomotion modulates the size of the HbT increase, but not the initial HbT decrease in the draining vein. For correlation analyses in 3a-b, p-values are taken from a Pearson's correlation (cor.test package RStudio) which tested for a linear relationship between the size of the locomotion event (AUC for 5s after onset) and the size of the HbT maximum or minimum peak (calculated from the maximum or minimum value reached in 5s after locomotion onset).

**Figure**	**Test**	**Test Statistic**	**95% CI**	**Degrees of freedom**	**P Value**
3a (Art maxpk)	Pearson's Correlation	t=14.48<br>r=0.28	0.249 - 0.323	2341	p<2.2e-16
3a (WV maxpk)	Pearson's Correlation	t=10.98<br>r=0.22	0.182-0.259	2341	p<2.2e-16
3a (DV maxpk)	Pearson's Correlation	t=7.49<br>r=0.15	0.113-0.192	2341	p=9.6e-14
3b (Art minpk)	Pearson's Correlation	t=-2.64<br>r=-0.05	-0.095–0.014	2341	p=0.0083
3b (WV minpk)	Pearson's Correlation	t=-2.23<br>r=-0.05	-0.086–0.00056	2341	p=0.0257
3b (DV minpk)	Pearson's Correlation	t=-0.02<br>r=-0.00039	-0.041-0.040	2341	p=0.9849

SR3: Figure 3: The amount of locomotion modulates the size of the HbT increase, but not the initial Hbt decrease in the draining vein. A linear mixed model was conducted for 3f which tested for a significant impact of vessel type (3 levels: artery, whisker vein, draining vein), locomotion group (2 levels: bottom or top 10% locomotion trials) or a vessel type * locomotion group interaction on HbT peak responses (maximum peak for artery and wv, minimum peak for dv). Vessel and locomotion groups were inputted as fixed factors, and animal ID as the random factor using the lmer package in RStudio.

**Figure**	**Mean**	**SD**	**Test**	**Test Statistic**	**Mean Square**	**Degrees of freedom**	**P Value**
3f (peak)	Art maxpk bottom	Art maxpk bottom 10%: 2.77	Linear mixed model	Vessel type F=1478.29,	Vessel type 10084.1, Locomotion	Vessel type 2, Locomotion	Vessel type p<2.2e-16, Locomotion

	10%: 4.61 Art maxpk top 10%: 9.49 WV maxpk bottom 10%: 2.06 WV maxpk top 10%: 4.61 DV minpk bottom 10%: -1.79 DV minpk top 10%: -2.56	Art maxpk top 10%: 5.11 WV maxpk bottom 10%: 1.52 WV maxpk top 10%: 3.19 DV minpk bottom 10%: 1.79 DV minpk top 10%: 2.21	(fixed factors: vessel type, locomotion group)	Locomotion group F=134.19, Vessel type * Locomotion group F=138.52	group 915.3, Vessel type * Locomotion group 944.9	group 1, Vessel type * Locomotion group 2	group p<2.2e-16, Vessel type * Locomotion group p<2.2e-16
Pairwise comparisons:	bottom 10% art - top 10% art p<.0001, bottom 10% art - bottom 10% dv p<.0001, bottom 10% art - top 10% dv p<.0001, bottom 10% art - bottom 10% wv p<.0001, **bottom 10% art - top 10% wv p=0.6273**, top 10% art - bottom 10% dv p<.0001, top 10% art - top 10% dv p<.0001, top 10% art - bottom 10% wv p<.0001, top 10% art - top 10% wv p<.0001, **bottom 10% dv - top 10% dv p=0.9254**, bottom 10% dv - bottom 10% wv p<.0001, bottom 10% dv - top 10% wv p<.0001, top 10% dv - bottom 10% wv p<.0001, top 10% dv - top 10% wv p<.0001, bottom 10% wv - top 10% wv p<.0001<br>**Non-significant comparisons denoted in bold font**						

SR4: Figure 3: The amount of locomotion modulates the size of the HbT increase, but not the initial HbT decrease in the draining vein. As well as conducting two-group comparisons using the Tukey post-hoc (emmeans package in RStudio) associated with the linear mixed model, we also analysed the maximum peak (for whisker artery and whisker vein) independently of the minimum peak in the draining vein. For the maximum peak we conducted a non-parametric two-way ANOVA (ggpubr package in RStudio) due to the data not being normally distributed (Shapiro-Wilks test: art- W=0.91, p=9.02e-7; wv- W=0.85, p=1.31e-9), and for the minimum peak in the draining vein we conducted a Wilcoxon rank sum test due to the data not being normally distributed (Shapiro-Wilks test: dv- W=0.88, p=4.92e-8).

**Figure**	**Mean**	**SD**	**Test**	**Test Statistic**	**Mean Square**	**Degrees of freedom**	**P Value**
3f (maximum peaks)	See SR3	See SR3	Non-parametric two-way ANOVA	Vessel Type F=89.44, Locomotion group F=142.97, Vessel type * Locomotion group F=11.47	Vessel Type 744.97, Locomotion group 1190.86, Vessel type * Locomotion group 95.53	Vessel Type 1, Locomotion group 1, Vessel type * Locomotion group 1	Vessel Type p<2.2e-16, Locomotion group p<2.2e-16, Vessel type * Locomotion

							group p=0.00083
Pairwise comparisons:	Art bottom – wv bottom p<0.0001, art bottom – art top p<0.0001, art bottom – wv top p=0.467, wv bottom – art top p<0.0001, wv bottom – wv top p<0.0001, art top – wv top p<0.0001						
3f (dv minimum peak)	See SR3	See SR3	Paired Wilcoxon-Signed Rank	W=2034		1	p=0.024

SR5: Figure 4: The meningeal vein shows a larger HbT decrease following locomotion onset, which unlike for the draining vein does not subsequently increase above baseline. Two-group comparisons were conducted to test for differences in HbT dynamics between draining and meningeal vessels. The assumptions of the unpaired t-test were assessed in GraphPad Prism using a Kolmogorov-Smirnov test of normality, and F test to compare the variances. For each figure plot (4c-f) the assumptions were violated, and the Mann-Whitney non-parametric alternative test was used.

**Figure**	**Mean**	**SD**	**Test**	**Test Statistic**	**Sum of Ranks**	**Degrees of freedom**	**P Value**
4d HbT Minima	dv: -2.358 mn: -3.971	dv: 1.952 mn: 3.063	Mann-Whitney test	U= 502017	dv: 1745859 mn: 1251717	1222	p<0.0001
4e HbT Normalised speed metric	dv: 0.9924 mn: 4.544	dv: 0.912 mn: 4.656	Mann-Whitney test	U= 216212	dv: 965912 mn: 2031664	1222	p<0.0001
4f HbT Area Under Curve	dv:0.7059 mn: -6.105	dv: 8.44 mn: 12.73	Mann-Whitney test	U= 471610	dv: 1776266 mn: 1221310	1222	p<0.0001

SR6: Figure 4: The meningeal vein shows a larger HbT decrease following locomotion onset, which unlike for the draining vein does not subsequently increase above baseline. Due to the significant difference in the amount of locomotion between draining vein and meningeal groups, the impact of locomotion on the Hbt minima was assessed using a linear regression. Neither the draining vein nor the meningeal vessel showed a significant correlation between locomotion and the HbT minimum (see is the slope significantly non-zero). Furthermore, there was no significant difference between the vessel groups on the relationship between locomotion and the HbT minima (see are the slopes equal). The intercepts were significantly different between groups as the minimum peak negative values were larger in the meningeal group.

**Figure**	**Test**	**Is the slope significantly non-zero?**	**Are the slopes equal?**	**Are the intercepts equal?**

4c Locomotion / HbT Minima Scatter	Linear Regression	dv: F(1,1222)=0.3038, p=0.5816<br>mn: F(1,1222)=0.5123, p=0.474	F(1,2444)=0.0944, p=0.7587	F(1,2445)=241.3, p<0.0001

SR7: Figure 5: The locomotion-induced draining vein HbT decrease is enhanced in mixed mice. A linear mixed model was conducted for 5e-f which tested for a significant impact of vessel type (3 levels: artery, whisker vein, draining vein), genotype (4 levels: group 1, group 2, group 3, group 4) or a vessel type * genotype interaction on HbT peak responses (HbT maximum peak for e, HbT minimum peak for f). Vessel and genotype groups were inputted as fixed factors, and animal ID as the random factor using the lmer package in RStudio.

**Figure**	**Mean**	**SD**	**Test**	**Test Statistic**	**Mean Square**	**Degrees of freedom**	**P Value**
5e (HbT max peak)	Art: AD: 6.21 WT: 7.75 ATH: 8.00 MIX: 7.81 WV: AD: 2.92 WT: 4.11 ATH: 3.88 MIX: 3.75 DV: AD: 2.00 WT: 2.43 ATH: 2.08 MIX: 1.49	Art: AD: 4.17 WT: 4.76 ATH: 5.02 MIX: 5.10 WV: AD: 2.38 WT: 2.97 ATH: 3.11 MIX: 3.07 DV: AD: 2.34 WT: 2.53 ATH: 2.34 MIX: 2.32	Linear mixed model (fixed factors: vessel type, genotype)	Vessel type F=1569.7, Genotype F=2.372, Vessel type * Genotype F=12.556	Vessel type 15832.3, Genotype 23.9, Vessel type * Genotype 126.6	Vessel type 2, Genotype 3, Vessel type * Genotype 6	Vessel type p<2.2e-16, Genotype p=0.086, Vessel type * Genotype p=3.9e-14
Pairwise comparisons:	**Due to high number of comparisons, only non-significant results reported:** art AD - art WT p=0.128, art AD - wv WT p=0.103, art AD - wv ATH p=0.190, art AD - art MIX p=0.208, art AD - wv MIX p=0.288, dv AD - dv WT p=0.989, dv AD - dv ATH p=0.999, dv AD - wv MIX p=0.129, wv AD - wv WT p=0.435, wv AD - wv ATH p=0.473, wv AD - dv MIX p=0.986, wv AD - wv MIX p=0.856, art WT - art ATH p=0.999, art WT - art MIX p=1.00, dv WT - dv ATH p=1.00, dv WT - wv ATH p=0.266, dv WT - dv MIX p=0.998, dv WT - wv MIX p=0.702, wv WT - dv ATH p=0.208, wv WT - wv ATH p=1.00, wv WT - wv MIX p=1.00, art ATH - art MIX p=1.00, dv ATH - dv MIX p=0.999, dv ATH - wv MIX p=0.643, wv ATH - wv MIX p=1.00						

	**Note that for interpreting vessel type * genotype interactions attention was focused on within vessel type comparisons that were significantly different between genotype (i.e. for artery max peak group 1 and group 3 differed p=0.02).**						
5f (HbT min)	Art: AD:-1.37 WT:-0.71 ATH:-0.82 MIX:-0.82 WV: AD:-0.98 WT:-0.81 ATH:-1.26 MIX:-1.29 DV: AD:-2.48 WT:-2.92 ATH:-2.54 MIX:-3.74	Art: AD: 2.03 WT: 1.73 ATH: 2.14 MIX: 2.01 WV: AD: 1.13 WT: 1.32 ATH: 2.49 MIX: 1.45 DV: AD: 1.98 WT: 2.84 ATH: 2.72 MIX: 2.63	Linear mixed model (fixed factors: vessel type, genotype)	Vessel type F=626.83, Genotype F=0.927, Vessel type * Genotype F=22.684	Vessel type 2549.11, Genotype 3.77, Vessel type * Genotype 92.25	Vessel type 2, Genotype 3, Vessel type * Genotype 6	Vessel type p<2e-16, Genotype p=0.438, Vessel type * Genotype p<2e-16
Pairwise comparisons:	**Due to high number of comparisons, only non-significant results reported:** art AD - art WT p=0.838, art AD - wv WT p=0.949, art AD - art ATH p=0.999, art AD - wv ATH p=1.00, art AD - art MIX p=0.999, art AD - wv MIX p=1.00, dv AD - dv WT p=0.757, dv AD - dv ATH p=0.999, dv AD - wv ATH p=0.146, dv AD - wv MIX p=0.442, wv AD - art WT p=1.00, wv AD - wv WT p=1.00, wv AD - art ATH p=1.00, wv AD - wv ATH p=0.941, wv AD - art MIX p=1.00, wv AD - wv MIX p=0.971, art WT - wv WT p=0.999, art WT - art ATH p=0.999, art WT - wv ATH p=0.658, art WT - art MIX p=1.00, art WT - wv MIX p=0.808, dv WT - dv ATH p=0.998, dv WT - dv MIX p=0.555, wv WT - art ATH p=1.00, wv WT - wv ATH p=0.835, wv WT - art MIX p=1.00, wv WT - wv MIX p=0.916, art ATH - wv MIX p=0.993, dv ATH - dv MIX p=0.135, dv ATH - wv MIX p=0.119, wv ATH - art MIX p=0.997, wv ATH - wv MIX p=1.00, art MIX - wv MIX p=0.150. **Note that for interpreting vessel type * genotype interactions attention was focused on within vessel type comparisons that were significantly different between genotype (i.e. for dv min peak group 1 and group 4 differed p=0.01).**						
